# Chicago Medical Society: Meeting July 19

**Published:** 1886-09

**Authors:** 


### CHICAGO MEDICAL SOCIETY.

*Stated Meeting, July 19, 1886.*—E. J. DOERING, M. D., President, in the chair.

PROFESSOR D. A. K. STEELE read a paper on

### THE DIFFERENTIAL DIAGNOSIS OF SCROTAL TUMORS.

For diagnostic or clinical purposes tumors of the scrotum may be divided into two general classes, viz.: reducible and irreducible—reducible tumors including all herniæ (except strangulated), varicocele, and congenital hydroceles. Irreducible tumors of the scrotum would include all tumors connected with the testicle, all hydroceles (except congenital), and strangulated hernia. Tumors of the tegumentary surface of the scrotum, such as inflammatory œdema, elephantiasis and epithelioma, are usually so characteristic in their history as to offer no special impediments to a correct diagnosis.

Scrotal hernia may be mistaken for:

1. Hydrocele of tunica vaginalis, cord, or encysted hydrocele.
2. Sarcocele of the testicle, simple, tuberculous, cystic or malignant.
3. Varicocele.
4. Hæmatocele.
5. Bubo.
6. Undescended testis.

In scrotal hernia, as a rule, the tumor is soft and doughy to the touch, light weight, smooth and regular, painless unless inflamed or strangulated, of sudden advent from above downwards, resonant on percussion, fills the inguinal canal, has cough-impulse, gurgles, is of normal color, opaque, may exist on either side, the spermatic cord is concealed, does not fluctuate, aspiration is negative, bowels may be embarrassed. It can be reduced unless the hernia is strangulated or incarcerated.

1. (a) In hydrocele of the testicle the tumor is ovoid or pyriform, develops slowly from below upward, is firm, tense and elastic, fluctuates, is translucent, dull on percussion; is irreducible; spermatic cord is neither concealed or displaced; the inguinal canal is empty; bowels unaffected; aspiration reveals fluid.

(b) In hydrocele of the cord the difference is that the tumor is circumscribed, encysted, and sometimes reducible, situated usually near the epididymis, but returns after reduction irrespective of position.

(c) In congenital hydrocele the fluid disappears completely within the peritoneal cavity by compression of the tumor for a short time.

2. (a) In sarcocele of the testicle the tumor is usually hard and resistant, heavy, often nodular and irregular; painful; grows slowly; dull or flat on percussion. The inguinal canal is empty; no impulse on coughing; bowels unaffected; irreducible; no auscultatory sounds. Simple sarcocele is chronic orchitis. Both the epididymis and body of the gland are affected. The cord is usually thickened. Abscess of the organ may occur. It is caused usually by an injury followed by inflammatory deposits.

(b) Tubercular sarcocele is met with most frequently in early manhood, and may occur in any constitution; in the strong and robust as well as the weak and cachectic; and although often associated with tubercularization of other organs, it is common enough to find the tuberculous nidus in the epididymis, not as a sequence of gonorrhœal inflammation or some slight injury followed by inflammatory infiltration—as was formerly believed—but as a coincident.* The progress is slow and insidious. The gland at first moderately enlarges with little or no pain, the hypertrophy being especially marked in the globus major. Presently the outline of the tumor becomes craggy or nodulated, and circles around the testicle from behind forward in the form of a crescent. After several months this adventitious tissue exceeds in size the testicle proper, and then it begins to soften at points, and one or more abscesses burst and discharge a thin shreddy pus. The vas deferens is greatly enlarged.

(c) In syphilitic sarcocele or gummata the history of the patient guides us in the diagnosis. Also we find that the body of the gland is usually the seat of the infiltration which takes place in the connective tissue between the tubuli seminiferi, the epididymis undergoing little if any enlargement. The cord and vas deferens are unaffected. There is little or no tenderness, and the peculiar sensation elicited by squeezing a healthy testicle is absent. The tunica albuginea is very greatly thickened. Hydrocele is a frequent complication, and tapping is often required to establish a diagnosis.

*Cystic tumors* of the testis closely resemble hydrocele, and differ chiefly in being opaque instead of tianslucent.

---

* The bacilli tuberculosis which were formerly probably disporting in the blood in a state of innocuous desuetude, now finding a fertile soil in the increased vascularization of the part, proceed to inhabit, multiply and take possession of the part to its ultimate destruction—just as other microbes have an affinity for the epiphysis of long bones where the blood current is sluggish.

Aspiration should be practiced before pronouncing positively upon their character.

Cancer of the testicle primarily invades the body of the gland, and almost invariably assumes the encephaloid form. Most observers doubt the existence of other varieties of malignant disease in this organ. The development of the disease is rapid. The patient has a sensation of weight, pain and dragging in the testis, the scrotum becomes distended, reddish or purplish, and the superficial veins are seen to be enlarged. The skin adheres to the gland, ulceration occurs, fungus protrudes, the inguinal glands are secondarily involved, and the patient by this time presents the characteristic cancerous cachexy.

3. In varicocele the tumor is knotty and irregular, like a bag of worms; bluish in color; most frequent upon the left side; increases in size upon the application of heat; develops gradually; is dull on percussion; fluctuation doubtful; spermatic cord not affected; inguinal canal not involved. No cough-impulse. It reduces spontaneously by any position that favors increased venous return, but returns immediately when the patient stands up, notwithstanding pressure at the ring. There is weight and dragging in the scrotum.

4. In hæmatocele the advent is sudden; usually traumatic in origin; grows from below up if spontaneous; fluctuates until coagulation occurs; is at first soft, and hard after coagula form. It is pyriform in shape; ecchymotic; irreducible; heavy; dull on percussion. The spermatic cord is unaffected, and the inguinal canal empty. There is often pallor and prostration from loss of blood. The bowels are unaffected.

5. Bubo is seldom mistaken for a scrotal tumor, and it is unnecessary to name differential points.

6. An *undescended testicle* is painful, and pressure upon it yields a peculiar sickening sensation. It is found wanting upon the side occupied by the tumor, and the scrotum is imperfectly developed upon the same side. It is sometimes mistaken for a bubonocele.

PROFESSOR CHRISTIAN FENGER agreed with Professor Steele that gonorrhœa in itself has nothing to do with tuberculosis; that it will not cause local tuberculosis of the epididymis any more than a trauma will cause local tuberculosis anywhere else in the body. When there is tuberculosis in the body somewhere, and consequently tubercle-bacilli in the current of the blood, then it may be that the local inflammation from gonorrhœal epididymitis, just as extravasation of the blood from a trauma, favors the accumulation of tubercle-bacilli in those places. Occasionally local tuberculosis will commence in an inflamed epididymis, but in a large number of these cases of scrotal tuberculosis there has really been no gonorrhœic epididymitis preceding.

PROFESSOR W. T. BELFIELD thought that too much stress had been laid upon the history as a means of differential diagnosis between syphilitic and other enlargements of the testicle; when affirmative, the history is a valuable factor; when negative, the diagnosis must be made without regard to the history. He mentioned a case where there was not only no history of syphilis, but also a nodular enlargement of the epididymis with but slight increase in the size of the testis —features usually characteristic of tuberculosis; yet the patient's age (31) argued so strongly against tuberculosis that it was determined to try syphilitic remedies before proposing excision; the effect was immediate and complete. Primary tuberculosis of the genital organs is evidently an infection from within—hæmatogenous. The idea of local infection through intercourse with a subject of uterine or ovarian tuberculosis is of course fanciful; indeed, very many of these patients are boys just beyond puberty who rarely have had intercourse. The growth of the tubercle bacilli implies some local or general predisposition of the individual; one factor in this predisposition is evidently found in the conditions developed during the rapid growth of a part. Thus in childhood the epiphyses of long bones frequently afford a nidus for the growth of the parasite, while the rudimentary sexual organs are never affected primarily; but when with puberty the genital organs begin a rapid development, they are especially prone to become the site of the infection.

The tuberculous testicle should be excised, provided the prostate and seminal vesicles are apparently healthy, to remove the chances of further infection. While it is of course possible that there may be other and undiscovered foci of infection in various parts of the body—lymphatic glands, lungs or elsewhere—yet castration not only removes one, perhaps the only tuberculous nidus, but especially diminishes the danger of infection of the prostate and seminal vesicles—a necessarily fatal as well as agonizing affection. Tuberculosis of the lungs is sometimes arrested by proper climatic and hygienic conditions; but tuberculosis of the prostate never, so far as we know, by either hygienic, medical or surgical means.

DR. McARTHUR had little to say in regard to the differential diagnosis. In regard to scrotal tumors there was a point upon which he would like to have light, viz.: whether Dr. Fenger would have the Society believe that tubercular testis is always the result of general tuberculosis; that is, that the tubercular bacilli are brought in the blood to the point where the local inflammation occurs. He had privately asked Dr. Belfield if such were the case, what would be the advantage of an early removal? He had removed a testicle that happened to be a tubercular one. The man was 35 years of age, in apparently robust health, with no evidence of pulmonary or kidney affection. Dr. McArthur advised the removal of the testicle and performed the operation. On microscopic examination all the characteristics of tubercular testicle were found; within two months and a half the man died of acute tuberculosis.

PROFESSOR CHRISTIAN FENGER said that fifteen years ago nobody would have thought of extirpating a tuberculous testicle, because we knew that after the abscess has opened and discharged for a long time, such persons, if put under favorable circumstances, given cod-liver oil, sent to the seashore or country, will recover. So far as general tuberculosis resulting from a tuberculous testicle was concerned, only about 25 per centum of the patients, he thought, would become subject to general tuberculosis. In cases where the miliary tubercles were limited to the testicle, and all other parts of the body not as yet invaded, extirpation should be performed. It is a legitimate operation now, but has only become so within the last five years. It of course has to be limited to cases where, so far as we can find out, the tubercle is local in the epididymis. If the tuberculosis has invaded the vas deferens, it is difficult to see what good extirpation of the testicle could do except as a mere local measure. Another point in tuberculosis of the testicle makes us desire if possible to eradicate the local tuberculosis and thereby perhaps prevent the tuberculosis affecting the bladder. The patients are subject to most terrible suffering for a year or two before death when they get tuberculosis of the bladder. He did not think tuberculosis could be called local here except if it could be transmitted by cohabitation with a woman having tubercles of the uterus, much in the same way as the microbes of gonorrhœa are transferred, and this mode of infection is not proved as yet. The tubercles in the epididymis must originate in the same way as local tuberculosis in the bones, by arresting any accumulation of tubercle bacilli already preëxisting in the general circulation. He did not think it possible that, in the strict sense of the word, any of these tubercles could be called local.

PROFESSOR G. C. PAOLI did not claim to be a specialist, but had seen many tumors of the testicles and scrotum. Tumors of the scrotum often arise in connection with diseases of the prostate glands, and with strictures. He had seen tumors of the testicle produced by affections of these organs. Another thing that gives trouble in practice is neuralgia of the testicles, which is very difficult to diagnose because the only symptom we have is the severe pain in the testicle. Another kind of tumor which has not been mentioned is scirrhous, and it is often difficult of diagnosis. Many that have been diagnosticated as scirrhous have been nothing but chronic orchitis. He once saw a testicle extirpated in the belief that it was scirrhous, but it proved to be a case of chronic inflammation of the testicle.

DR. J. L. GRAY read a paper on

LIGATION OF THE VERTEBRAL ARTERIES IN EPILEPSY.

He said: The ligation of the large vessels furnishing blood to the brain, for the specific purpose of relieving epilepsy, was first proposed in 1831, he believed by a Dr. Brown, of Calcutta, though, it has been stated, that prior to that date, the operation was performed successfully in this country by a surgeon upon his colored servant.

The operation for ligating the vertebral arteries to control epileptic seizures was first advised by Dr. Wm. Alexander, of Liverpool.

The vertebral arteries, with their branches, furnish the blood supply of the medulla, pons, cerebellum and posterior third of the cerebrum, while the carotids furnish the blood supply for the anterior two-thirds of the cerebrum.

The relation between the sympathetic nerves and vertebral artery is very close indeed, and during its course through the transverse processes of the vertebræ, the artery is surrounded so closely by fibres of the sympathetic that they cannot be isolated without injury to its coats.

It seems to be generally admitted that the real seat of local disease in epilepsy is in the great group of centres in the medulla, more especially the vaso-motor centres and the regions closely related thereto, as, for example, the convulsive centre pointed out by Nothnagel.

The morbid excitations which arouse these centres to unhealthy action must come from regions outside the centres themselves; that is, the local disease may be present for years, and yet no fit occur, provided stimuli of sufficient intensity to rouse the centres to unhealthy action are prevented from reaching them.

In the vast majority of cases of epilepsy, the excitations which give rise to the attack start in some part of the digestive tract from the mouth down, or in the genito-urinary apparatus. These excitations are propagated through the sympathetic up into the medulla, there to excite to unhealthy action the abnormally irritable centres situated in its depths. Excitations may come from other regions of the body, as, for example, from the brain itself.

There are two theories which have been advocated in support of the benefit to be derived from ligating the vertebral arteries for the relief of epilepsy. The one which was first proposed, is that held by Alexander. In brief, his theory is, that the operation is of benefit in modifying the circulation by diminishing the quantity of blood sent to the diseased and hyper-sensitive centres.

There are several objections to the adoption of this theory of the modification of the blood supply to the parts of the brain and central nervous system in which the disease is.

After ligation of both vertebrals, the part of the brain which had been supplied with blood by these arteries receives its blood supply from the carotids through the posterior communicating arteries of the circle of Willis. It is probable that, within a very short time, these communicating arteries dilate to such an extent as to allow of nearly as great a blood supply to the posterior third of the cerebrum as it received before the ligation of the arteries. It does not, as Alexander leads us to suppose, take months or years to have the collateral circulation established. If this were the case, we should observe those symptoms which accompany cerebral anæmia; but no evidence of anæmia is observable immediately after the operation.

If the benefit derived be due to diminishing the blood supply to the brain, the operation ought to prove of as great benefit in those cases where the excitation starts in the cortex and is propagated downward into the medulla as in those cases where the excitation arises in some region outside the central nervous system,—say in the digestive tract. But such is not the case. Thus far, it is believed, the operation has proved of no benefit whatever in those cases of epilepsy due to cortical disease of the brain, or where the attack is produced by strong emotional excitement of any kind.

The second theory is that proposed by Dr. Jewell: that the operation is of benefit not merely in diminishing the blood supply to the parts in question, but in preventing morbid excitations from below reaching the medulla by way of the sympathetic, the fibres of which are included in the ligature around the vertebral.

The question as to the best place to ligate the artery is not an unimportant one. Dr. Alexander has operated at the sixth cervical vertebra.

If the artery is ligated at this point the communication between the middle and superior cervical ganglion of the sympathetic and the medulla is not severed. This allows morbid excitations to reach the medulla through the carotid and cavernous plexuses and the pneumogastrics.

The high operation, so called, between the axis and atlas, seems to be the preferable one if we wish to sever, in the most complete manner, communication through the sympathetic between the peripheral nervous system and the excitable centres in the medulla.

Dr. Alexander has reported twenty-one cases, three have been quite well for nearly a year, nine others so free from fits for such a period of time that it may be said a cure would result, and eight are improved to such an extent that the operation is justified were no better results ever obtained. One case, a little girl, died from septic poisoning.

In Chicago the operation has been performed seven times. One case cured, one improved, one no improvement, one died, and in the three other cases time enough has not elapsed since the operation to give any report.

The operation thus far has not in this country been performed a sufficient number of times to allow any sound conclusions to be drawn. The reports of Dr. Alexander are certainly encouraging, giving, as they do, the results in the most intractable cases possible to imagine. From all the evidence at hand, we seem to be justified in drawing the following conclusions:

1. Ligation of the vertebral arteries should take its place as a recognized procedure in the treatment of certain cases of epilepsy.

2. The operation should be confined to those cases in which the exciting causes of the attacks come from some region outside the brain.

3. The arteries should be tied as high up as practicable, and the ligature should include all the fibres of the sympathetic accompanying the vessel.

4. Where the side of the brain which is first invaded by the disease can be determined, the artery of that side should be ligated.

5. Where the invasion of the disease is apparently bilateral, both vertebrals should be ligated.

6. This operation should not be done as a substitute, but as an aid to other forms of treatment for the relief or cure of epilepsy.

PROFESSOR D. R. BROWER, in opening the discussion, thanked Dr. Gray for his very able paper so full of interest and scientific importance, and said the case which he reported to the State Medical Society two years ago is now not quite so favorable. Indeed the child has practically relapsed into the same condition she was in at the time of the operation. For two or three months the change for the better in the child's condition was very manifest. In that case the ligation was made at one side. The intention was to have both arteries ligated, but such symptoms of collapse came on that it was deemed unwise to pursue the operation further, and there being so little benefit, the parents refused to have another operation. He thought with Dr. Gray that enough is before us favorable to the operation to recommend it in cases where everything else fails, and he would be disposed to press the operation in those cases where there seems to be some special disorder of the circulation of the medulla. He thought the trouble about the treatment of epilepsy is that we have included under this name a great many diseases. He was sure there are a great many kinds of epilepsy, and he thought we do not know how to differentiate these different kinds, therefore our treatment must be to a great extent imperfect. There seem to be cases of epilepsy that are confined to the medulla. The history of some cases led him to suppose that the medulla is the seat of the disease, and in such cases it seems that the operation might prove a benefit. He did not believe that the circulation is established throughout the circle of Willis nearly so rapidly as Dr. Gray would have us believe. The circle of Willis is made up of minute arteries. In this particular case, there was a manifest and profound impression made upon the area of distribution of the vertebral artery for weeks, and he did not believe the collateral circulation was speedily established. He had no doubt that this operation would do good in some cases, just as any other profound impression will if made upon the nervous system; the cutting and destruction of the connections through the various plexuses is beneficial, so is any extensive counter irritation. A seton added to other treatment that was only partially successful sometimes caused a wonderfully better result to follow. He had a case under observation in which the removal of a cicatrix has resulted in wonderful improvement in the patient's condition, whether due to the extensive counter irritation produced by the operation or removal of cicatrix he did not know. He believed the operation justifiable in cases that resisted the ordinary treatment. He was an earnest advocate of the surgical treatment of epilepsy, the best results he had seen in this disease having followed the use of the surgeon's knife. Quite a number of cases had come under his observation in which the cause of the affection seemed to be in the ovarian region, and surgical interference was followed by better results than from treatment by medicine. He thought the treatment of epilepsy by pills and powders one of the most unsatisfactory things that can be undertaken, and the proportion of cases benefited by drugs exceedingly small.

PROFESSOR CHRISTIAN FENGER asked as to Alexander's operation, whether he ligated at once on both sides. The first time Professor Fenger ligated an artery the question presented itself if it would not be dangerous to ligate them both at the same time. He ligated the arteries at Dr. Jewell's request and consequently did not take responsibility for ligating both at once. That question of course presents itself, and is truly interesting to see that the double simultaneous ligation of the vertebral arteries has never given rise to local brain symptoms. On the other hand, when we ligate a common carotid in case of epilepsy, we always have in mind the danger which may exist of emollition, from local cerebral enemia, and possibly death in a certain number of cases. The operation on the vertebral arteries is far preferable to the ligation of the carotid, if it should prove that there is no danger by the ligature of the vessels both at one time. As to the manner of operating: Alexander operated down low before the artery enters the vertebral canal, but according to Dr. Jewell's view it is necessary to ligate the vertebral arteries high up. He looked over the books of surgery and found that the method of ligating between the occiput and the atlas, and between the latter and the epistrofous, had been done on the cadaver, but not on living subjects. The reason why the vertebral artery had been ligated in former times was either for aneurism or wounds. There are very few instances on record, and the wounds and aneurisms were very dangerous cases, and Fischer in the German surgery of Billroth and Lurcke, published in 1880, said that the ligature of the vertebral artery between the occiput and atlas and between the atlas and the axis is practically impossible. On the cadaver it can be done as well between the occiput and atlas as the atlas and axis, but the operation is much easier between the atlas and axis. To ligate between the occiput and the atlas Professor Fenger considers almost practically impossible on the living subject. The ligature between the atlas and the axis can be done. He knew that Andrews ligated there, but the cases on record are few. There has not as yet been any injury to the artery, but if we should tear the artery so that we could not get hold of vessel enough to ligate above and below, we would have much the same condition as in a traumatic aneurism of the artery. Warren, of Boston, who wrote his Memoirs of Surgery twenty years ago, gives one case of wounded artery that got well by compression, by filling up the wound, but in most cases we know how little effective the plugging of an arterial wound is. Of thirty-four wounds of the vertebral artery only five recovered. Four of these were treated by plugging or local applications; in one only the ligature between occiput and atlas was performed by him in 1881 and the case published in *Gaillard's Journal*, July 1882. He was afraid when taking up the vertebral artery of the possibility of wounding it before it is ligated.

DR. C. W. PURDY asked Dr. Gray whether the operation was followed by diabetes, as would be expected from an operation of that kind. It was pointed out in 1849 by Claude Bernard, that traumatic injuries to this tract of the brain gives rise to diabetes in numerous instances, and since that time the course of the diabetic influence has been pretty satisfactorily mapped out. It is said to leave the brain by vaso-motor filaments with the vertebral arteries going as far as the third cervical ganglion, passing along with and surrounding the subclavian arteries, and reaching the upper and middle dorsal. A section of these nerves at any place until we reach the upper dorsal ganglion causes diabetes. Strange to say, section of the splanchnics does not cause diabetes, but we would expect to find traumatic diabetes as a result of this operation. He thought that diabetes as a result of the operation would be only temporary.

DR. HAROLD N. MOYER thanked Dr. Gray for the manner in which he had presented the subject, and especially for the indications which he laid down for the operation. Dr. Moyer had uniformly bad results from the treatment of epilepsy by any operative procedure. He had never recommended an operation for trephining that was followed by good results. He had once recommended the removal of the ovaries, and it was done, but was not followed by a good result. In one case coming under his observation a man was trephined three times; the first time it was followed by a cessation of the epilepsy for eighteen months; the second, for a year; the last by no cessation. He had found medication quite as bad; he had tried the bromides according to Hammond's method, without good results. During the last two years he had treated about one hundred cases of epilepsy and in only one case had there been any real benefit. He thought he should recommend this operation, and hoped it might not be as serious as one would be led to suppose from the deep seat of the artery, and the extensive destruction of tissue that must be made to reach it.

PROFESSOR D. R. BROWER said that at the discussion which followed his paper at Springfield, a gentleman present announced that this operation was first performed in this country sixty years ago by an army surgeon, stationed in St. Louis, upon a negro man who was his servant. The man recovered, but the case was never published. If this is so, an American surgeon was the first to essay the operation.

DR. J. L. GRAY, in closing the discussion, said that he had not had an opportunity, except in one case, to examine the urine, and in that there was a slight quantity of sugar for about a week. From all the literature he was able to examine the first operation was performed in 1831 by Dr. Brown.

---

CHICAGO MEDICAL SOCIETY.

